# Simultaneous occurrence of triple teeth and double teeth in primary dentition: A rare case report and review of the literature

**DOI:** 10.1002/ccr3.2876

**Published:** 2020-04-29

**Authors:** Marianne Lagarde, Anne‐Laure Bonnet, Nina Douangmala, Marina Traing, Elisabeth Dursun

**Affiliations:** ^1^ Faculty of Dental Surgery Paris University Montrouge France; ^2^ Henri Mondor Hospital Créteil France; ^3^ Innovative Dental Materials and Interfaces Research Unit (UR 4462) Montrouge France; ^4^ Charles Foix Hospital Ivry‐sur‐Seine France; ^5^ Orofacial Pathologies Imaging and Biotherapies Laboratory (UR 2496) Montrouge France

**Keywords:** double teeth, fused teeth, micro‐computed tomography, triple teeth

## Abstract

This paper reports the case of a 3‐year‐old male patient with triple teeth in the right maxillary incisor region and double teeth in the left mandibular incisor region. He had pre‐existing medical conditions. The triple teeth were extracted and examined using micro‐computed tomography. A literature review was performed to discuss this abnormality.

## Graphical abstract

Even if occurrence of triple teeth is very rare, early detection is crucial to prevent complications. Use of sealants to prevent caries development and monitoring exfoliation to avert malposition of permanent teeth are the key preventive measures.

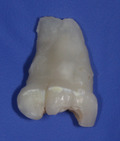

## 1 INTRODUCTION

Dental abnormalities such as fusion or gemination have been described in both dentitions. Fused teeth correspond to the union of two or three normal tooth germs (synodontia), or one or two normal tooth germs and one supernumerary tooth. Depending on when it happens during tooth development, the fusion can be complete or incomplete; the pulp chamber and root canal may be joined or separated. When fusion occurs after crown completion, the teeth are united only by the cementum; this is called concrescence.^1^, ^2^ Gemination is the failure of attempted tooth‐germ cleavage with incomplete formation of two teeth, usually with one pulp chamber, a single root, and a common pulp canal. Twinning represents complete formation of two nearly identical teeth, one normal and one supernumerary tooth, but fused as one, usually with a single root and a single pulp canal.^1^, ^2^ In the case of three tooth entities, the terms “triple tooth,”“triple teeth,” or “triplication” are used and have the advantage of covering all types of unions.^3^ In fact, the clinical aspect of fusion or gemination of normal or supernumerary teeth is similar.

The prevalence of triple teeth in primary dentition is rare (0.02%)^4^ and shows a predilection for the male sex and Asian populations.^5^ It occurs more frequently in the upper arch than in the lower arch. A number of etiological hypothesis have been suggested: Close developing tooth buds, insufficient space in the dental arch, and physical pressure or trauma can cause contact between tooth germs^6^ that results in necrosis of the epithelial tissue that separates tooth germs and leads to fusion; genetic factors (dominant autosomal heredity)^7^; and disturbances in the prenatal period or environmental factors such as viral infection during pregnancy, intake of thalidomide, and lack of vitamins.^8^ However, none of them proved satisfactory.

Shilpa and Nuvvula classified triple teeth into two types and subtypes.^9^ Type I corresponds to fusion with three pulp chambers and three root canals, which includes type Ia: fusion of two normal teeth with a supernumerary tooth and type Ib: fusion of three normal teeth. Type II corresponds to fusion with two pulp chambers and two root canals, which includes type IIa: combination of one geminated tooth and a supernumerary tooth, and type IIb: combination of one geminated tooth and a normal tooth. However, it is difficult to determine the type even with intraoral radiography.

This paper aimed to report a rare case of a young patient presenting large triple teeth in the right maxillary incisor region and double teeth in the left mandibular incisal region, which has not been reported in the literature before, and further discuss it by a comprehensive literature search on triple teeth.

## 2 CASE PRESENTATION

A 3‐year‐old male patient of Cambodian origin was referred for extraction of an unusual right maxillary incisor after a traumatic injury. According to his mother, there were no such anomalies in the other family members. The anamnesis revealed that the child was born premature and actually presented with delayed growth, hyperlaxity, and cerebellar atrophy. Moreover, his built was small for his age. These symptoms led to genetic exploration of syndromic diseases, but with no remarkable findings.

**FIGURE 1:**
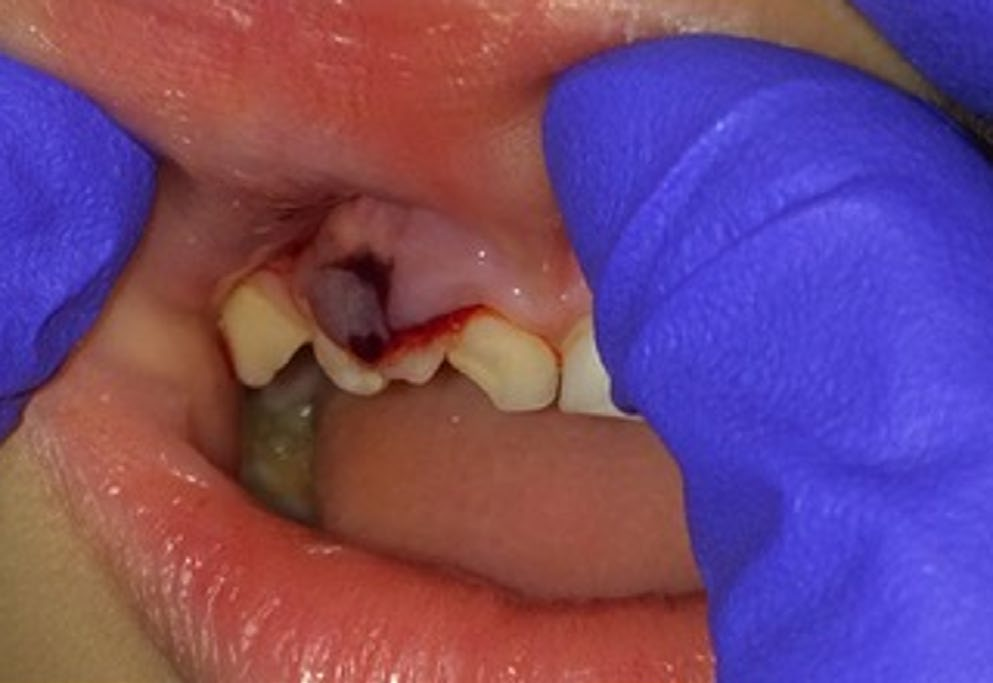
Intraoral view of the maxillary triple teeth and the gingival laceration

**FIGURE 2:**
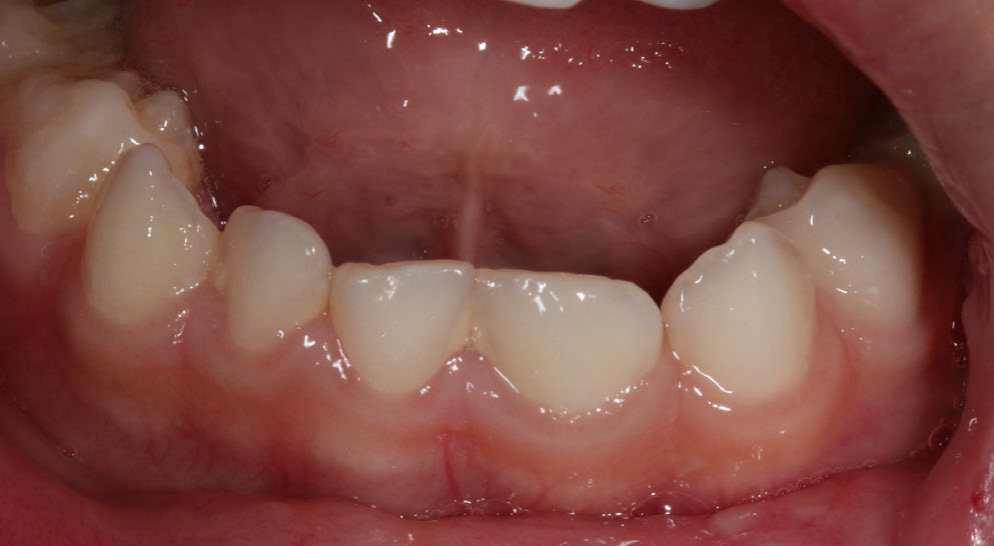
Intraoral view of the mandibular double teeth

The extraoral examination did not show any alterations. Intraoral examination revealed a gingival laceration next to a decayed triple tooth, in which was a double crown in place of the maxillary central incisor fused with the lateral incisor (Figure 1). These triple teeth were affected by a large carious lesion at the junction between the double crown and an incipient carious lesion in the groove between the double crown and the lateral incisor crown. These teeth presented no mobility or fracture. In the region of the right central incisor, a submucosal abscess was observed, suggesting infected pulp necrosis. Moreover, the left mandibular central and lateral incisors appeared fused, with a unique but larger crown (Figure 2). No other findings were reported.

**FIGURE 3:**
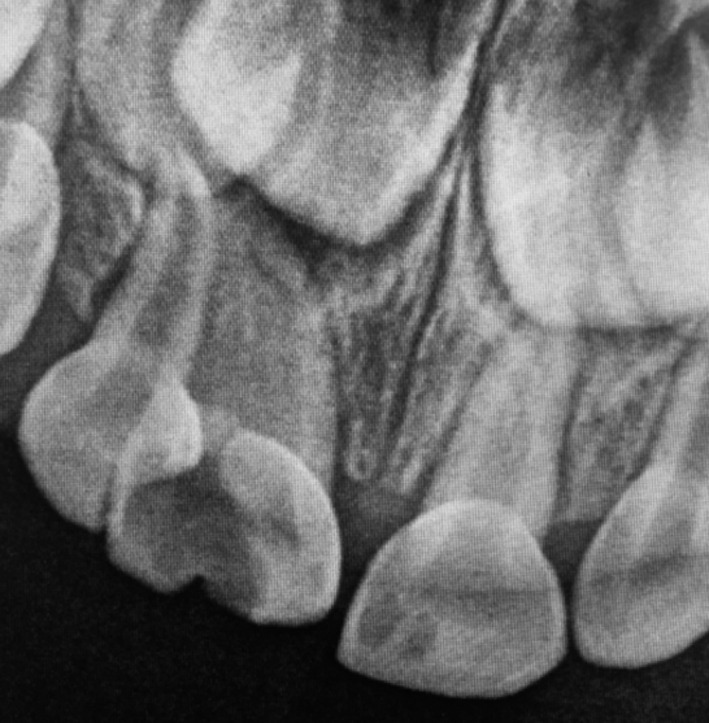
Radiographic image of the triple teeth

An intraoral periapical radiograph of the triple teeth revealed two distinct structures, an upper right incisor with a possibly unique pulp chamber (difficult to confirm because of the carious lesion) and a large root canal (possibly dividing into two canals in the middle‐third), and a lateral incisor with separate pulp chamber and separate root canal (Figure 3). It also showed a radiolucent area around the apex of the large central incisor, but the lateral incisor seemed unaffected. It was not possible to take a radiograph of the double teeth due to noncompliance of the patient.

**FIGURE 4:**
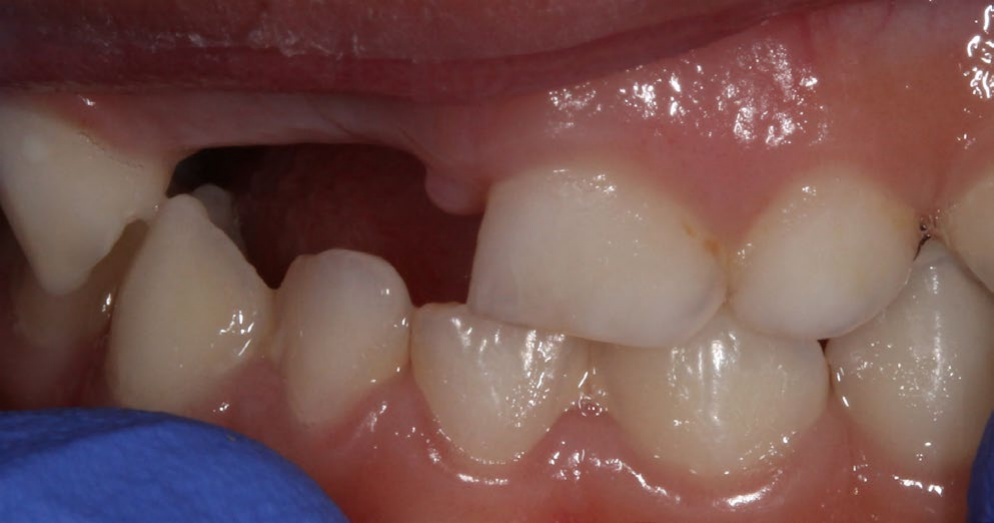
Intraoral view at 1‐y follow‐up

The diagnosis was gingival laceration due to trauma and abscess on the triple teeth due to an advanced carious lesion. Because of the difficulty in performing root canal treatment in such teeth and the poor cooperation of the young patient, extraction of the triple teeth was planned. Due to the difficulty in extracting such teeth and for the patient's comfort, this procedure was performed under nitrous oxide/oxygen inhalation. To prevent functional, esthetic, and phonetic problems, the missing teeth should have been replaced with a transitional partial denture. However, the replacement was not possible at this stage due to insufficient cooperation by the patient. A 1‐year follow‐up showed good healing (Figure 4). It was however not possible to perform a radiograph to check the underlying tooth germs, because of insufficient cooperation.

**FIGURE 5:**
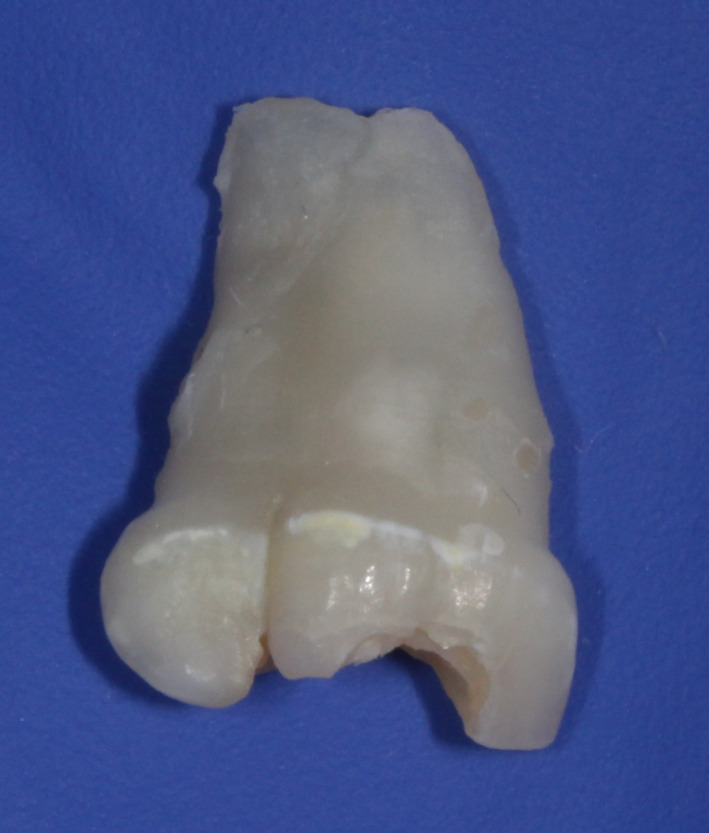
The extracted triple teeth

**FIGURE 6:**
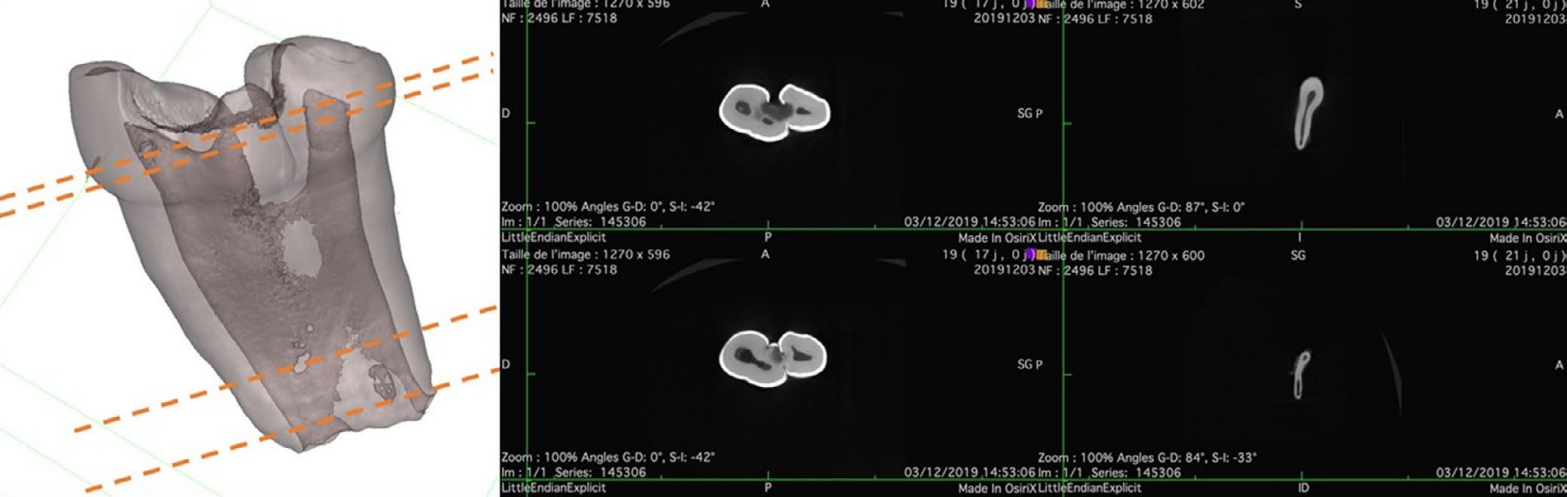
The micro‐computed tomography image of the triple teeth

The extracted teeth had three separate crowns and roots conjoined from the crown to the apex and possibly from the incisal edge to the apex in the large central incisor (Figure 5). Macroscopically, almost no root resorption was detected. The teeth were analyzed using micro‐computed tomography to obtain a three‐dimensional model and a two‐dimensional cross‐sectional slice (Figure 6). It showed two separate pulp chambers in the large central incisor, which were joined together at the cervical area, and one root canal. The lateral incisor presented separate pulp chamber and root canal, but its root canal was connected to the root canal of the large central incisor. These observations suggested gemination of the central incisor fused with the lateral incisor (type IIb of the classification by Shilpa and Nuvvula).

## 3 DISCUSSION

To our knowledge, coexistence of triple teeth and double teeth in two different arches has never been reported. To address this issue, an extensive literature review was conducted to identify all reported cases of triple teeth in the English language. A search was performed on the PubMed database using keywords related to triple teeth and primary dentition according to the following equation search: (triplication OR "triple teeth" OR "triple tooth" OR "three teeth" OR triplicated OR "three tooth fusion") AND (primary OR decidual OR deciduous OR temporary). It was manually completed with the references of the selected articles and a search on Google Scholar in order to find nonindexed publications. Inclusion criteria were all types of articles reporting or discussing triple teeth in primary dentition, written in English language, published up to December 2019, and availability of the full text. A table was created to collect details regarding age and sex of patients, teeth implicated, patient origin, familial history, medical context, diagnostic tools, radiographic interpretation, resorption trouble/delay of eruption, absence of successional tooth, other dental anomalies, and treatment performed.

All the articles are listed in Table 1.

**TABLE 1:** Literature data

Author, date	Patient's age and sex	Teeth implicated	Origin	Familial history	Medical context	Diagnostic tools	Radiography interpretation	Resorption troubles/delay of eruption	Absence of successional tooth	Other dental anomalies	Treatment performed
Gultekin et al^10^	5‐y‐old female	81, 82, sny	Probably Turkish	None	Noncontributory	Periapical radiograph, panoramic radiograph, CBCT	Fusion of 81 and 82 with a sny tooth (three separate pulp chambers joining in one pulp canal)	None	None	None	Regular follow‐up until normal exfoliation
Jeong et al^11^	Case 1: 1‐y, 7‐mo‐old male Case 2: 1‐y, 5‐mo‐old male	Case 1: 51, spy, 52 Case 2: 81, sny, 82	Case 1: probably Korean Case 2: probably Korean	Not mentioned	Not mentioned	Case 1 and Case 2: Intraoral periapical radiography	Case 1: Fusion of 51 and 52 with a sny tooth (two separate canals and pulp chambers for 51 and 52, sny tooth between them with root in developmental state) Case 2: Pulp chamber structure was obvious, but a canal structure was not clear for the sny tooth. The two primary teeth had clear canals	None	Case 1: Agenesis of 12 Case 2: None	Case 1: None Case 2: two mesiodens at maxilla, next to 11 and 21	Case 1: Pulpotomy Case 2: Extraction
Mallikarjun et al^12^	9‐y‐old male	72, sny, 73	Probably Indian	Noncontributory	Noncontributory	Periapical radiography, postavulsion histological examination with stereomicroscope	Fusion of 72 and 73 with a sny tooth (gemination of 72 fused with 73)	Slow root resorption, 31, 32 already erupted	None	22 and 12 in labial version	Extraction
Nagaveni et al^13^	7‐y‐old male	81, 82, 83 and 71, 72	Probably Indian	Noncontributory	Premature birth	Intraoral periapical radiography	Fusion of 81,82 and 83 and fusion of 71 and 72 (separate pulp chamber and root canals in both)	None	Agenesis of 42	None	Regular follow‐up until normal exfoliation
Thakkar et al^14^	Case 1: 5‐y‐old male Case 2: 5‐y‐old male	Case 1: 61, 62, sny Case 2: 51, 52, sny	Probably Indian	Not mentioned	Not mentioned	Case 1: Intraoral periapical radiography, CBCT Case 2: Intraoral periapical radiography	Case 1: Fusion of 61 and 62 with a sny tooth (three distinct crowns, one root with two equidistant grooves, but distinct root canals) Case 2: Fusion of 51 and 52 with a sny tooth (separate pulp chambers and root canals)	None	None	None	Case 1: Extraction (abscess) + transitional partial denture Case 2: Restorative treatment (decay)
Shanthraj et al^15^	7.5‐y‐old female	71, sny, 72	Probably Indian	Not mentioned	Not mentioned	Intraoral periapical radiography	Fusion of 71 and 72 with a sny tooth (all alongthe crown and the root: each tooth with distinct pulp chambers and root canals)	Yes, but resorption at 3 mo follow‐up	None	None	No treatment, root resorption until exfoliation
Juneja et al^16^	9‐y‐old male	61, sny, 62	Probably Indian	Noncontributory	Noncontributory	Periapical and occlusal radiography, postavulsion radiography, histological examination	Fusion of 61 and 62 with a sny tooth (three separate pulp chambers and root canals)	Slow root resorption, 21 palatally erupted.	None	21 palatally erupted, in Crossbite, 22 unerupted with space deficiency	Extraction, composite inclined plane on opposing mandibular teeth to correct the crossbite
Yadav et al^17^	10‐y‐old male	51, sny, 52	Probably Indian	None	Mentally challenged	Periapical radiography, histological examination with stereomicroscope	Fusion of 51 and 52 with a sny tooth (separate pulp chambers, separate root canals merging in the apical third)	Resorption only of the 52 (presence of 11)	Agenesis of 12	11 palatally erupted, in crossbite	Extraction (tooth retained)
Shilpa et al^9^	5‐y‐old male	61, 62, sny	Probably Indian	Noncontributory	Noncontributory	Occlusal radiograph	Fusion of 61 and 62 a with sny tooth	None	Agenesis 22	None	Regular follow‐up
Sharma et al^18^	7‐y‐old male	61, 62, sny	Probably Indian	Noncontributory	Noncontributory	Intraoral periapical radiograph, histological examination	Concrescence of 61 and 62 with a sny tooth (separate pulp chamber and root, union by cemental part)	Resorption only of the 61 (presence of 21)	None	21 palatally erupted	Extraction (tooth retained, palatal eruption of 21)
Babaji et al^19^	6‐y‐old male	81, sny, 82	Probably Indian	Noncontributory	Noncontributory	Intraoral periapical radiograph	Fusion of 81 and 82 with a sny tooth (separate pulp chamber and root canals)	Eruption delay (presence of 31 and 32)	Agenesis of 41	None	Recall examination until exfoliation
Mohapatra et al^20^	10‐y‐old male	51, sny, 52	Probably Indian	Not mentioned	Noncontributory	Intraoral periapical and occlusal radiograph, histological examination with stereomicroscope	Fusion and gemination (three separate crowns with separate pulp chambers at the crown, three joined roots with separate pulp canals at the middle third, progressively joined to form a common apical canal, dentinal fusion)	Eruption delay (presence of 21)	Agenesis of 12	Midline diastema	Extraction (permanent retained), removal prosthesis
Schultz‐Weidner et al^21^	4‐y‐old male	51, sny, 52 and 61, sny, 62	Thai	None	Noncontributory	Intraoral periapical radiography	Fusion of 51 and 52 with a sny tooth; fusion of 61 and 62 with a sny tooth (both with separate pulp chambers and root canals)	No root resorption	None	None	Opening chamber, then extraction, and transitional partial denture
Prabhakar et al^22^	6‐y‐old male	51, sny, 52	Probably Indian	Not mentioned	Not mentioned	Intraoral periapical radiography, histological examination	Fusion of 61 and 62 with a sny tooth (separate pulp chambers and root canals, fusion at enamel and cementum, only)	None	None	None	Extraction, then removal of prosthesis
Erdem et al^23^	2‐y‐old male	61, sny, 62	Probably Turkish	Not mentioned	Noncontributory	Intraoral periapical radiography, light microscope	Fusion of 61 and 62 with a sny tooth (separate pulp chambers and root canals, fusion by dentin and cement)	None	Agenesis of 22	None	Extraction (abscess) + transitional partial denture
Aguilo et al^24^	Case 1: 3‐y‐old female Case 2: 2‐y‐old male	Case 1: 61, sny, 62 Case 2: 51, sny, 52	Caucasian	None	Noncontributory	Intraoral periapical radiography, post‐avulsion radiography, histological examination, CT images	Case 1: Fusion of 61 and 62 with a sny tooth (separate pulp chambers and root canals, fused in apical) Case 2: Fusion of 51 and 52 with a sny tooth (separate pulp chamber and root canals, fused in the middle for two, then separate again)	Case 1: None (physiological resorption) Case 2: None	Case 1: None Case 2: Agenesis of 12	None	Extraction (Case 1: trauma and Case 2: abscess)
Rao^25^	6‐y‐old female	61, 62, sny	Probably Indian	Father with similar teeth	None	Intraoral periapical radiography, orthopantomogram	Fusion of 61 and 62 with a sny tooth (separate pulp chambers and root canals)	None (physiological resorption)	Agenesis of 22	Diastema in maxillary and mandibular arch	Restoration of decay teeth and seal the deep grooves
Mochizuki et al^26^	2‐y‐, 8‐mo‐old female	52, 51, 61	Japanese	None	None	Orthopantomogram, intraoral occlusal radiography	Fusion of 52, 51, and 61 (separate pulp chambers, root canal fused ¾th of the way from apex, one root)	Not mentioned	Underdeveloped 12, fused with 11	Width and length of dental arch less than the Japan national average	Sealant therapy, fluoride application, and monitoring
Riesenberger et al^27^	2‐y‐, 8‐mo‐old male	61, 62, sny and 51, 52	Not mentioned	Father with “double baby teeth”	Asthma	Intraoral occlusal radiography	Fusion of 61 and 62 with a sny and fusion of 51 with 52	None	None	None	Extraction of 61, 62, and the sny tooth (abscess); Extraction of 52 and restoration of 51
Trubman et al^28^	3‐y‐old male 6‐y‐old male	Case 1: 81, 82, sny Case 2: 81, 82, sny	Case 1: “black” Case 2: “white”	Not obtainable	None	Intraoral periapical radiography	Case 1: Gemination 81, fusion 82 Case 2: Gemination 81, fusion 82 (Both cases: 81 with one root canal and two crowns, 82 with one crown and one root canal)	None	Case 1: None Case 2: not determinable	None	Monitoring
Knapp et al^3^	6.5‐y‐old female	61, 62 and sny	“White”	None	None	Intraoral occlusal and periapical radiography, post‐avulsion radiography	Gemination of 61 or early fusion with sny tooth, and fusion with 62 (central and mesial elements with shared pulp chamber and separate root canal, distal element with distinct pulp chamber and root canal)	Eruption delay	Lower development rate of 22 as compared to 12	None	Monitoring, followed by extraction (tooth retained) and space maintainer
Dhooria et al^29^	10‐y‐old male	61, 62, sny	Probably Indian	None	None	Intraoral periapical radiography, postavulsion radiography	Fusion of 61 and 62 with a sny tooth (separate pulp chambers and root canals)	Slow root resorption, delay of resorption	None	Successor teeth already erupted (21 and 22)	Extraction
Burley et al^30^	Case 1: 4‐y‐, 10‐mo‐old female Case 2: 2‐y‐, 11‐mo‐old male	Case 1: 61, 62, sny Case 2: 61 62	Not mentioned	Sister and brother with similar dental features	None	Case 1: intraoral periapical radiography Case 2: intraoral occlusal radiography	Case 1: Fusion of 61 and 62 with a sny tooth (common radicular pulp) Case 2: Fusion of 61 and 62 (common radicular pulp)	None	None	Case 1: Misplaced 21, sny 22, conical teeth Case 2: conical teeth	Extraction
Long^31^	7‐y‐old male	71, 72, sny	Not mentioned	Not mentioned	Not mentioned	Post‐avulsion radiography	Fusion of 71 and 72 with a sny tooth	Slow root resorption, lingual eruption of 32	Agenesis of 31	32 lingually erupted	Extraction

Abbreviations: CBCT, cone beam computed tomography; CT, computed tomography; mo, months; sny, supernumerary; y, years.

Our compilation corroborates with the literature data that females are less commonly affected (seven cases), and the maxillary and left side of the arch are more commonly affected.^9^ Fusion of three normal teeth is rare (two cases),^13^, ^26^ with only one case showing affected teeth on both right and left sides.^26^ All other cases showed fusion with a supernumerary tooth or gemination. In addition, the association of triple teeth with double teeth is also rare (three cases) with respect to the same arch.^13^, ^21^, ^27^ Ours is the first case showing triple teeth associated with double teeth in two different arches.

It has been suggested that triple or double teeth are more frequent in Mongolian or Asian populations.^5^ Our search reported more than half of the cases were of probable Indian origin,^9^, ^12^, ^13^, ^14^, ^15^, ^16^, ^17^, ^18^, ^19^, ^20^, ^22^, ^25^, ^29^ that is, cases reported by Indian authors. However, there is a bias in the publications selection. In fact, Indian authors are better in writing English as compared to Japanese or Korean authors for examples. However, it should be noted that few European or American authors have reported such cases. In addition, one third of these cases^3^, ^12^, ^13^, ^15^, ^16^, ^17^, ^18^, ^20^, ^29^, ^31^ included children aged > 6 years, indicating a late screening of triple teeth.

Familial or medical history is not often reported. Only three cases reported familial history^25^, ^27^, ^30^ and three cases reported medical history.^13^, ^17^, ^27^ However, the number of cases reporting familial history may be lower than the actual number, because familial history relies on the memory of parents or other family members. Even if these may not be common etiologies, they could be possible aggravating factors.^13^ In fact, the only patient who reported a premature birth was one of the two cases showing triple teeth and double teeth, similar to our patient. Thus, premature birth could be an aggravating factor. Moreover, one of these two cases combined familial and medical history.^27^

Most studies only use intraoral periapical or occlusal radiographs, limiting the distinction between fusion and gemination as well as the relationship and proximity between the triple teeth and adjacent and underlying teeth. Panoramic radiographs help in better examination of the entire dental situation, especially to detect potential agenesis of underlying permanent teeth. In fact, 10 cases^9^, ^11^, ^13^, ^17^, ^19^, ^20^, ^23^, ^24^, ^25^, ^31^ reported missing successional teeth and one case reported presence of two mesiodens.^11^ The cone beam computed tomography (CBCT) avoids image distortions and superimpositions, allowing easy observation of root canal and precise determination of resorption areas. However, it is difficult to perform panoramic radiography in very young patients and CBCT is irradiating. Only three cases underwent panoramic radiography,^10^, ^25^, ^26^ while CBCT was used in two cases.^10^, ^14^

Furthermore, several cases reported crossbite or malalignment,^12^, ^16^, ^17^, ^18^, ^29^, ^31^ underlying the importance to monitor tooth resorption and its timely exfoliation.

In most cases, monitoring was implemented^3^, ^9^, ^10^, ^13^, ^15^, ^19^, ^26^, ^28^ or extraction was indicated,^11^, ^12^, ^14^, ^16^, ^17^, ^18^, ^20^, ^22^, ^23^, ^24^, ^27^, ^29^, ^30^, ^31^ as in our case. Only three cases had undergone restoration procedure^11^, ^25^, ^27^ and one case underwent pulpotomy.^11^ Because of the complexity of the root canal system, reliable root canal treatment is almost impossible; thus, all efforts should be made to avoid carious lesions. Sealants should be placed in the grooves of the occlusal surfaces, followed by regular monitoring. Only two cases reported sealing of the grooves,^25^, ^26^ whereas 10 cases were monitored. In cases of deep pulpal involvement or periapical lesions, extraction is inevitable. In cases of delayed exfoliation, extraction is also recommended to avert malocclusion.

## 4 CONCLUSION

Fused teeth are initially asymptomatic and rarely seen in children. Aside from esthetic concerns, they can develop carious lesions in their grooves, pulpal inflammation, or abscesses. Root canal therapy is not a reliable treatment. Fused teeth could also lead to delayed exfoliation, resulting in space problems, occlusal disturbances, and delayed eruption of the permanent successors. Therefore, early identification is crucial to implement preventive/simple restorative treatment followed by careful monitoring until exfoliation. In cases of delayed physiological root resorption, extraction at the age of normal exfoliation should be implemented to prevent late eruption of the permanent teeth.

## CONFLICT OF INTEREST

None declared.

## AUTHOR CONTRIBUTIONS

ML: treated the patient, conducted the literature search, and wrote the manuscript. AB: performed the 3D analysis and gave final approval. ND and MT: treated the patient and gave final approval. ED: directed the work, analyzed the literature search, and wrote the manuscript.
